# To Our Readers

**Published:** 1849-01-01

**Authors:** 


### TO OUR READERS.

---

The Journal of Psychological Medicine and Mental Pathology having reached the anniversary of its first appearance, we shall, no doubt, be expected to address a few observations to our intelligent, and, we have reason to hope, well-satisfied readers. The January of 1849 brings with it an agreeable epoch: it is to us the commencement of a new cycle of existence—the advent of a Second Volume; and, without arrogating to ourselves any undue degree of credit for the success we have achieved, we may fairly congratulate and thank our Contributors for the able support they have given us; our Subscribers, for the honour of their patronage; and our Readers—professional and unprofessional—for the attention and interest which they have manifested in the progress of the science which it is the special object of this Journal to record. And here it may not be irrelevant to remark, that when this publication was announced by a prospectus, which was not intended to herald its approach with any very great flourish of trumpets, numerous were the disparaging predictions which on every side assailed us. Such a journal, said one, never can succeed; it will be a jungle of metaphysical quiddities and weather-beaten sophisms—who will care to explore and entangle themselves in its tortuous and briery paths? The study of metaphysics (exclaimed another) has long since been exploded, and fail of necessity it must, for it will eventually be "gravelled for lack of matter." Last, not least, an anonymous oracle, wiser than the rest, vouchsafed a verbal hypercriticism on the title we had adopted. Psychological medicine! What can psychology have to do with medicine? How can the word pathology be applied to the different phases of mental aberration? These and a host of similar interrogatories literally besieged us; and the fair prospect before us was almost darkened with a cloud of gloomy prognostications—but we were not disheartened. We entertained a lively faith in the cause we had already espoused, and recognised the star in the ascendant which would guide us on in our course. The metaphysical "jungle" which perplexed the schoolmen of the middle ages, we knew had been already cleared. The paths of mental philosophy, we had ascertained, were open and plain, and could easily be explored by all who might be desirous of entering them. Nay, it is not true that metaphysical science, in so far as the study of the human mind is concerned, is so utterly neglected as those who have been inattentive to this department of knowledge are willing to believe. The recent publication of Reid's collected writings, with Notes and Addenda by Sir William Hamilton; the admirable and eloquent "Historical and Critical View of the Speculative Philosophy of Europe in the Nineteenth Century," by Morell; the "Philosophy of the Inductive Sciences," by Whewell; the "History of the Philosophy of the Mind," by Blakey; sufficiently prove that the spirit of intellectual philosophy in England is not yet extinct. Indeed, the simple fact that the works of Plato, Schlegel, Ritter, &c., are in the course of publication, in cheap monthly volumes, would alone seem to indicate that mental science, instead of being repudiated and despised, is becoming daily more popular.

The study of the human mind having attained so distinct a speciality, the word psychology has been appropriately applied to this branch of science. The phenomena of the mind were formerly investigated without any reference to the principles of physiology and pathology. In the early history or infancy of these sciences, this isolation could scarcely be avoided; but now that our views are more comprehensive, the mutual relation which exists between them is better understood, and psychology is recognised to have not only a theoretical, but a practical connexion with medical science.

We are indebted to Reil and Hoff'bauer, in Germany, for having established this association between these sciences. Reil was an accomplished anatomist; Hoff'bauer, who had imbibed the principles of Kant, was an acute metaphysician; both perceived the mutual relation which existed between the result of their investigations, and their united labours gave rise to the first Medico-Psychological Journal that was published in Germany,

(1806 1808.) This journal, however, was chiefly philosophical. The increasing and continually extending interest felt on the subject of lunacy, which proceeded from individual institutions, and from the different states, soon called for a more decided medical organ; and, in 1818, another journal of psychological medicine was published under the editorship of Nasse. To this succeeded (in 1829—1838) Freiderich's Magazine, and another periodical of a similar character was edited by Jacobi and Nasse. In 1841, Damerow issued an address in Berlin, calling upon the psychological physicians of Germany to establish a journal, which should have special reference to public institutions for the insane; and, in 1844, the project was ably carried into effect. In Paris, the Annales Medico-Psychologiques, edited by Baillarger, Cerise, and Longet, and supported by contributions from Bellingeri, Bouchet, Brière de Boismont, Falret, Mitivié, Parchappe, Lelut, Foville, Royer-Collard, Voisin, and other eminent collaborateurs, has appeared for the last six years, every second month, and enjoys an extensive circulation, not only in Europe, but in America. Recently also a Medico-Psychological Society has been established in Paris, the object of which is to advance the knowledge of Mental Pathology and the accessory sciences. In the United States also there has appeared for several years a Monthly Psychological Journal, under the title of the "American Journal of Insanity," edited by officers of the New York State Lunatic Asylum. This is an able record of the progress of medical psychology among our Transatlantic friends.

Accordingly, the objections which were in the first instance urged against the establishment of this Journal are, by the facts we have adduced, completely overruled. Not only have we found that there is no deficiency of *matériel* for such a journal, but that the supply greatly exceeds our limits, and that the most difficult task is frequently the compression and abbreviation of the articles selected, the value of which depends, in our estimation, not on their theoretical ingenuity, but upon the practical application of the doctrines they propound. All philosophical speculations, conducted upon inductive principles, are legitimate, but it is the application of them only which is the ultimate test of their validity; this is the only criterion of their truth. How, indeed, we may ask, is it possible to treat philosophically and successfully the mind in a state of aberration, if we are ignorant of the laws of our mental constitution? It is true that we have emerged from that age of intellectual barbarism, when every indication of insanity was regarded as a sign of demoniacal possession—when solitary confinement, chains, bolts, stripes, and other instruments of physical torture were had recourse to; but let us not congratulate ourselves too hastily, and view our present system of treatment with too much self-satisfaction.

The transition from darkness to light is palpable; the release of the limbs from instruments of cruel restraint and oppression is a relief to suffering humanity; the mild and gentle voice of persuasion, in many cases, is more effective than tones of harsh and imperative command. The entire system of moral treatment has undergone a revision, amounting to a complete regeneration; but we must not suppose that we have yet attained the *ultimatum* of progression. Much remains yet to be done; and if in Germany, France, and, we may add, in Italy, periodicals of psychological medicine are deemed of importance to represent the interests of institutions for the insane, and to promote the advancement of mental pathology, how obvious it is that such a journal in England must be of equal consequence. Our experience—indeed we may say, the success which the Journal of Psychological Medicine has met with during the last year—is to us a sufficient evidence that it supplies a *desideratum* which had previously existed; and the encouragement we have received both at home and abroad, inspire us with a confidence and an energy, which we feel assured will sustain us in realizing the success which we originally anticipated. We therefore, with elated and cheerful feelings, enter upon the second volume of the Journal. We are happy in being enabled to announce that we have secured the additional assistance of many able and eminent contributors—and have made several new arrangements with correspondents on the Continent for the earliest publication of any discoveries or intelligence which may advance the progress of Medical Psychology.

Sussex House, Hammersmith,
1st January, 1849.

